# A Salutary Tale—Glargine insulin and cancer risk

**DOI:** 10.3332/ecancer.209.ed5

**Published:** 2009-07-27

**Authors:** Peter Boyle

**Affiliations:** International Prevention Research Institute, 95 cours Lafayette, 69006 Lyon, France

Insulin glargine is a long-acting insulin analogue, authorised in the European Union (EU) as *Lantus* and *Optisulin*, for the treatment of adults, adolescents and children aged six years or above with diabetes, when treatment with insulin is required.

On 26th June 2009, four articles were published on-line in *Diabetologia*: one each from Germany [Hemkens et al, 2009], Sweden [Jonasson et al, 2009], Scotland [SDRN, 2009] and England [Currie et al, 2009] which were interpreted in an accompanying Editorial [Smith and Gale, 2009] of demonstrating that the risk of cancer was increased among patients being treated with glargine insulin for their type II diabetes.

Following review of all available information on a possible relationship between insulin analogues, in particular insulin glargine, and the risk of cancer, the European Medicines Agency’s Committee for Medicinal Products for Human Use (CHMP) concluded in a Press Release on 22nd July 2009 that the available data does not provide a cause for concern and that changes to the prescribing advice are therefore not necessary.

## Sequence of Events

This is a strange story which had the undoubted potential to cause significant distress to patients and their doctors and yet which all could have been avoided.

A manuscript apparently containing the first description of an increased cancer risk in diabetic patients taking glargine insulin was submitted to *Diabetologia* which was eventually sent to six reviewers, three of whom recommended that the paper be rejected [Smith and Gale, 2009].

The Editors, however, decided to publish the article and requested to some Administrative Database holders if they could confirm the findings in their database. This is extraordinarily unusual since it should have been expected that any such finding would be so important that the relevant authorities should have been informed first.

Previous to this, there had been no indication from *in vivo* or *in vitro* studies or in randomized trials that glargine insulin was associated with an increased risk of cancer. The hypothesis generated by the German study was that “the cancer incidence with glargine was higher than expected compared with human insulin” (Hemkens et al, 2009).

The hypothesis was tested in the three other studies with the following conclusions noted in the Abstracts of each of the studies:

“.....no statistically significant results were obtained [....] for the outcome all malignancies” [Jonasson et al, 2009].

“insulin glargine use was not associated with an increased risk of all cancers or site-specific cancers in Scotland”. [SDRN, 2009]

“Use of insulin analogues was not associated with increased cancer risk as compared with human insulin” [Currie et al, 2009].

It is evident that the hypothesis generated by the German study of Hemkens et al (2009) has not been confirmed in either of the three studies in which confirmation was sought. The hypothesis, therefore, should have been dismissed.

## Limitations of Observational Studies Conducted in Administrative Databases.

In my opinion, the situation should never have reached the point where an increased risk of cancer was a question for the public health. The German study was so obviously flawed from the methodological point of view that it should not have been published in the first place. It is a good rule of reviewing never to take the decision of the results presented in a manuscript, but to review critically the methodology on which any conclusions are based.

The German study of Hemkens and coworkers, shared with the other three studies the strengths and weaknesses of all observational studies conducted on prescription. Each of the four studies reported are large, have a prospective design and have the ability to assess risk in patients who use only one type of insulin. The studies have major weaknesses in terms of a relatively short duration of follow-up (2.4 years is the longest mean follow-up in the four studies), the total lack of control for bias by indication (BBI) and an inability to deal with the issue of dose in the statistical analysis. With so much key information missing about important cancer risk factors, there is little hope of dealing with the issue of bias by indication in these studies.

Large sample size never overcomes bias.

## Methodology of the Hypothesis-Generating Study (Hemkens et al, 2009).

The German study had some specific methodological problems.

Hemkens et al (2009) report the puzzling finding that when adjustment is made for age and gender, glargine is significantly protective against cancer risk and when dose is added to the list of factors entered into the model, the glargine is associated with a statistically significant increase in the risk of cancer.

This puzzling result has at least two origins: [1] overweight or obese patients are usually treated with higher insulin doses compared to normal weight individuals; and [2] the statistical problems induced by collinearity in regression models, when variables entered in the model are correlated between themselves. The consequence is the production of unstable results by the statistical model, with complete reversal of risks very possible.

More statistical issues undermine the interpretation of the conclusions of this study. The Cox proportional hazard model was used, thus assuming that variable had no time-dependent differences [Pocock and Smee, 2009]. This basic assumption of the Cox model was not met in this dataset as subjects taking insulin were often under treatment since much longer than subjects taking glargine. So, statistical outputs may just reflect inadequate use of a statistical method [Pocock and Smee, 2009]. In addition, that statistical models included all four types of insulin simultaneously, when three separate models should have been fitted for comparing aspart, lispro and glargine to insulin and statistical outputs may just reflect inadequate use of statistical.

In summary, this study is based on poor methodology and there are several key sources of bias and confounding which would influence the findings. The study shows no increased risk with glargine insulin although when adjustment is made for dose in inappropriate mathematical models, there appears an association with overall risk of cancer and this risk is only observed when glargine is the only insulin prescribed. The risk is no longer detected when patients taking glargine are also taking another type of insulin.

## Summary and Conclusions

The initial hypothesis generated by the German study (Hemkens et al, 2009) was not confirmed in either of the three confirmatory studies (Jonasson et al, 2009; SDRN, 2009; Currie et al, 2009). Dose-response association between insulin glargine and cancer was observed in the German registry (following a questionable statistical modelling approach) but not in the Swedish, Scottish, and United Kingdom registries. Results across different types of cancers were inconsistent. The inconsistencies and other limitations suggested that the results of these registries are not conclusive, and only raise some questions which they did not answer and may warrant further investigation.

This is a prime example of how studies with major potential for bias and severe methodological limitations, can result in unsound conclusions being drawn and cause anxiety among patients and their physicians unless the results are carefully interpreted. The study as it stood had sufficient obvious methodological flaws which should have argued strongly against publication.

There is no basis at present for an effect of glargine insulin acting as a risk factor for the development of cancer. However, these studies do raise questions which must be addressed in other studies based on a stronger methodology which could give some unambiguous results.

This storm resulted from an inadequate study with unsound methodology which could only lead to unsound conclusions being drawn. Unfortunately, when researchers rush to be “First” based on such poor quality data, they must remain aware of the collateral damage this can cause to many patients and their doctors.

## References

[1] Currie CJ, Poole CD (2009). Gale EAM: The influence of glucose lowering therapies on cancer risk in type 2 diabetes. Diabetologia.

[2] Hemkens LG, Grouven U, Bender R, Gunster C, Gutschmidt S, Selke GW, Sawicki PT (2009). Risk of malignancies in patients with diabetes treated with human insulin or insulin analogues: a cohort study. Diabetologia.

[3] Jonasson JM, Ljung R, Talback M, Haglund B, Gudbjornsdottir S, Steineck G (2009). Insulin glargine use and short-term incidence of malignancies: a population-based follow-up study in Sweden. Diabetologia.

[4] Pocock SJ, Smee L (2009). Insulin glargine and malignancy: an unwanted alarm. Lancet Oncology.

[5] (2009). SDRN Epidemiology Group: Use of insulin glargine and cancer incidence in Scotland: a study from the Scottish Diabetes Research Network Epidemiology Group. Diabetologia.

[6] Smith U, Gale EAM (2009). Does Diabetes Therapy influence the risk of cancer. Diabetologia.

